# A solid beta-sheet structure is formed at the surface of FUS droplets during aging

**DOI:** 10.1038/s41589-024-01573-w

**Published:** 2024-03-11

**Authors:** Leonidas Emmanouilidis, Ettore Bartalucci, Yelena Kan, Mahdiye Ijavi, Maria Escura Pérez, Pavel Afanasyev, Daniel Boehringer, Johannes Zehnder, Sapun H. Parekh, Mischa Bonn, Thomas C. T. Michaels, Thomas Wiegand, Frédéric H.-T. Allain

**Affiliations:** 1 Department of Biology, Institute of Biochemistry, ETH Zurich, Zurich, Switzerland; 2 Bringing Materials to Life Initiative, ETH Zurich, Zurich, Switzerland; 3 Max Planck Institute for Chemical Energy Conversion, Mülheim/Ruhr, Germany; 4 Institute of Technical and Macromolecular Chemistry, RWTH Aachen University, Aachen, Germany; 5 Department of Biomedical Engineering, University of Texas at Austin, Austin, TX USA; 6 Max Planck Institute for Polymer Research, Mainz, Germany; 7 Department of Materials, ETH Zurich, Zurich, Switzerland; 8 Cryo-EM Knowledge Hub, ETH Zurich, Zurich, Switzerland; 9 Laboratory of Physical Chemistry, ETH Zurich, Zurich, Switzerland

**Keywords:** NMR spectroscopy, Protein aggregation, Biophysical chemistry

## Abstract

Phase transitions are important to understand cell dynamics, and the maturation of liquid droplets is relevant to neurodegenerative disorders. We combined NMR and Raman spectroscopies with microscopy to follow, over a period of days to months, droplet maturation of the protein fused in sarcoma (FUS). Our study reveals that the surface of the droplets plays a critical role in this process, while RNA binding prevents it. The maturation kinetics are faster in an agarose-stabilized biphasic sample compared with a monophasic condensed sample, owing to the larger surface-to-volume ratio. In addition, Raman spectroscopy reports structural differences upon maturation between the inside and the surface of droplets, which is comprised of β-sheet content, as revealed by solid-state NMR. In agreement with these observations, a solid crust-like shell is observed at the surface using microaspiration. Ultimately, matured droplets were converted into fibrils involving the prion-like domain as well as the first RGG motif.

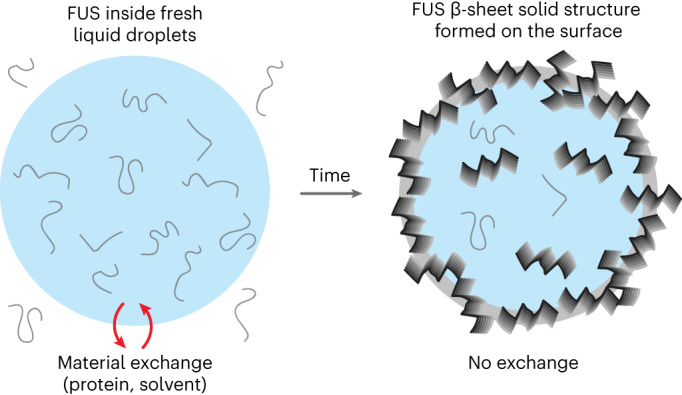

## Abstract (web summary)

NMR and Raman spectroscopies pinpoint the role of the protein droplet surface and RNA in the liquid droplet maturation mechanism of the FUS protein. A crust-like β-sheet structure is formed on the surface of FUS droplets during aging.

## Main

Research on the phase-separation behavior of biomolecules has exploded in recent years as gradually more cellular functions are found to rely on such phenomena^1,2^. Many proteins will phase separate from the aqueous environment to form an additional phase via a plethora of transient weak noncovalent interactions and may assemble into cellular membraneless organelles^3–5^. A now-commonly detected state is a liquid condensed phase, which enables rapid material exchange with the surrounding cytoplasm or nucleoplasm^6^. In vitro, this behavior can be reconstituted by the formation of liquid droplets. Interestingly, the liquid state of these entities, both in vitro and in vivo, may not be thermodynamically stable. Indeed, over time, some of these liquid droplets transition to a less-dynamic and often even solid-like state through a process known as maturation or aging^1,7^. Because this solid state has been linked to various neurodegenerative diseases, it is of great interest to understand the mechanism of this transition and the associated loss of the dynamic nature of liquid droplets. To date, no proposed model or mechanism posits how protein droplets transition to different states. In particular, which physical properties of the droplets allow for gradual solidification and how the atomic structure of the molecules influences the state of the matter are key questions that remain unanswered.

Here, we utilize the RNA-binding protein of FUS, which self-assembles in vivo under stress to form stress granules in the cytoplasm^8^. FUS, like other RNA-binding proteins, has previously been shown to phase separate in vitro, and the resulting liquid droplets have been reported to rigidify over time, with disease-related mutations promoting significantly faster maturation than in the wild-type^9–11^. Furthermore, a short segment of the N-terminal unstructured half of the protein has been shown to form amyloid fibrils after several days in vitro^12–14^ although a complete molecular pathway for this liquid-to-solid transition is not known. Here, we investigate the liquid-to-solid transition of FUS droplets stabilized in agarose using a combination of (solution- and solid-state) NMR spectroscopy, spatially resolved coherent Raman spectroscopy, electron microscopy and micropipette aspiration, and we reveal the molecular pathway for the FUS droplet maturation process.

## Results

### First days of FUS maturation studied by solution-state NMR

**Fig. 1:**
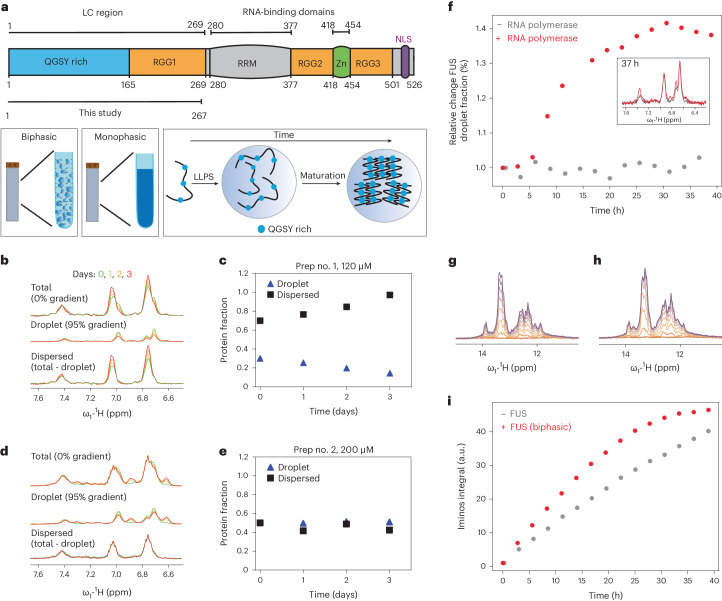
Evolution of the FUS phase fraction over time studied using solution NMR. **a**, Schematic representation of FUS protein domain organization. LC, low complexity; NLS, nuclear localization signal; RRM, RNA recognition motif; Zn, zinc finger. Definitions of biphasic and monophasic are also shown: biphasic contains liquid droplets, whereas monophasic is a single continuous dense phase as result of the sedimentation of droplets. The maturation process is the transition from liquid droplets to less-dynamic immobile species. **b**,**c**, Overlaid DOSY spectra at different time points at 120 μM (**b**) and the corresponding integrals for direct comparison (**c**). **d**,**e**, Overlaid DOSY spectra at different time points at 200 μM (**d**) and the corresponding integrals for direct comparison (**e**). At zero gradient strength the total population of FUS is visible, whereas at maximum gradient strength only the droplet phase is visible. The difference between the two reports is the dispersed fraction only. Panels **d** and **e** represent the two distinct modes of behavior observed in this study. **f**, Relative FUS droplet fraction change within 40 h in the presence and absence of RNA transcription, with the corresponding ^1^H NMR spectra shown in the inset. **g**,**h**, Overlay of ^1^H NMR spectra at different time points focused on the imino region in the absence (**g**) and presence (**h**) of FUS liquid droplets. Orange indicates 0 h changing progressively to magenta (40 h). **i**, RNA iminos integral progression over time indicating the faster kinetics of the biphasic sample. a.u., arbitrary units.

To study FUS droplet maturation, we prepared several biphasic FUS N-terminal domain (NTD) samples (Fig. 1a; residues 1–267) stabilized inside an agarose hydrogel, as described previously^15^. Briefly, highly concentrated protein stock is diluted in agarose containing warm buffer while still liquid. As the temperature drops and the sample solidifies, protein droplets are formed. The hydrogel acts as a cytoskeleton mimic that prevents sedimentation and maintains micrometer-size droplets, allowing us to study the droplet structure in vitro under conditions similar to physiologically relevant stress granules. Furthermore, agarose hydrogel enables FUS droplet stabilization without conformational perturbations^15^.

Proteins inside FUS liquid droplets diffuse at least 100 times more slowly than proteins outside^16^. This difference enables us to observe and quantify the protein fraction within liquid droplets (henceforth the condensed phase) using diffusion ordered spectroscopy (DOSY) NMR experiments^15^. Therefore, the difference between the condensed phase protein and the total protein enables us to quantify the amount of protein in the dilute phase.

**Extended Data Fig. 1:**
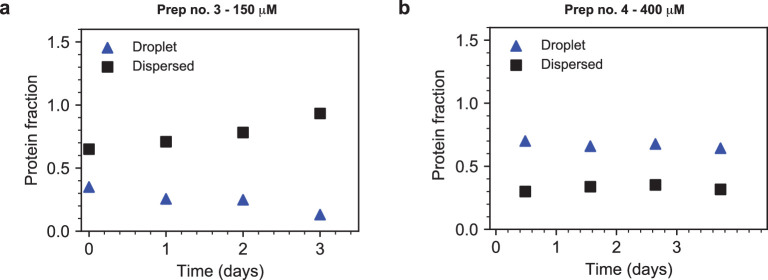
FUS fractions over time measured with different protein preparations and concentrations. a) At 150 μM of prep no. 3 droplet fraction simultaneously decays as the dispersed fraction increases. The two rates of change are equal as highlighted by their average which is stable at one. b) At 400 μM of prep no. 4 both droplet and dispersed fractions remained unaltered over time.

We undertook a series of DOSY experiments over 3 days using different FUS preparations and various protein concentrations (120–400 μM). Surprisingly, we observed two types of maturation behavior. In type 1 (two cases, corresponding to the lowest concentrations tested: 120 μM (case 1) and 150 μM (case 3)), we measured a decrease in the NMR signal corresponding to the condensed phase and a simultaneous increase in the dilute phase fraction (Fig. 1b,c and Extended Data Fig. 1a). Because we did not observe any obvious change in turbidity in the NMR tube, we presume that the droplets did not dissolve but rather their chemical environment changed. In type 2, at higher initial FUS concentrations (200 μM (case 2) and 400 μM (case 4)), we observed no significant evolution of the fractions over the first 3 days (Fig. 1d,e and Extended Data Fig. 1b).

**Extended Data Fig. 2:**
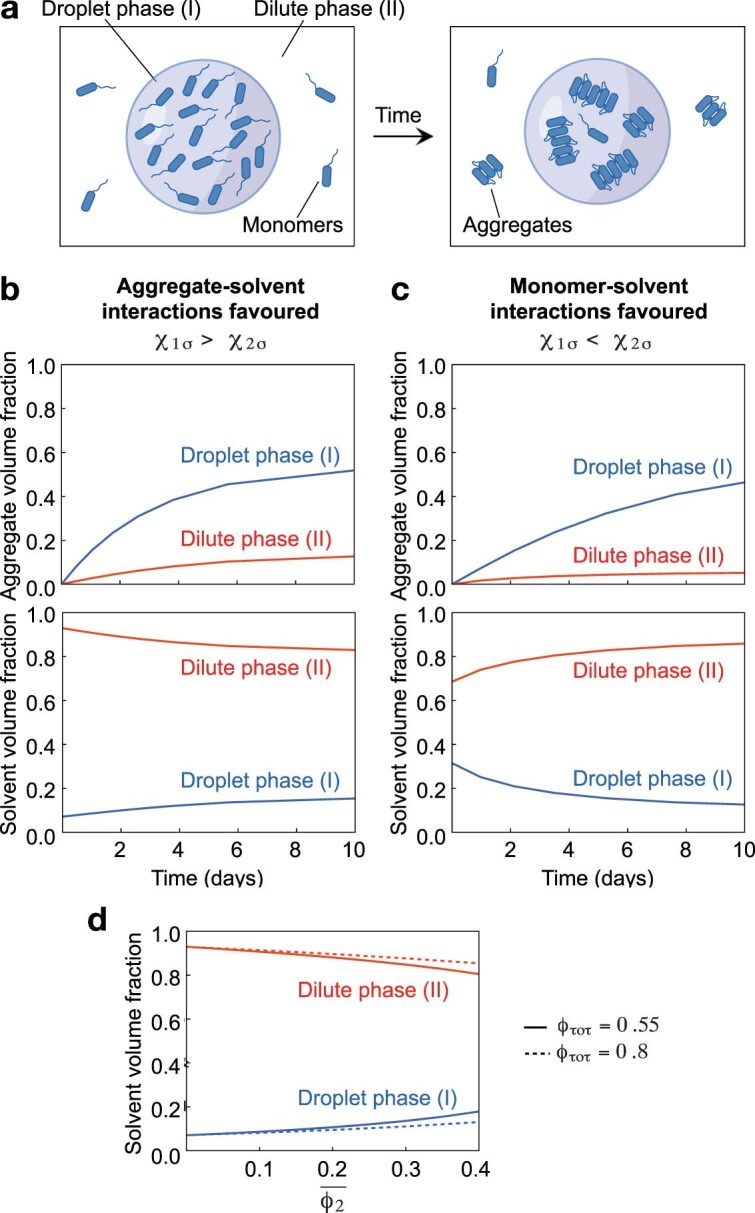
Solvent behavior during maturation. (a) Schematic representation of the model of FUS aggregation in coexisting phases. (b,c) Time evolution of aggregate and solvent volume fractions in the droplet (I) and dilute (II) phases for the case when (b) monomer-solvent interactions are disfavored over aggregate^−^solvent interactions (${\chi }_{1s} > {\chi }_{2s}$), or (c) aggregate-solvent interactions are disfavored over monomer-solvent interactions (${\chi }_{1s} < {\chi }_{2s}$). See Methods for parameters used in the plots. (d) Solvent volume fraction as a function of the total aggregate concentration formed during the reaction $\bar{{\phi }_{2}}$ for two different initial conditions, ${\phi }_{{tot}}=0.55$ (solid lines) and ${\phi }_{{tot}}=0.8$ (dashed lines). Simulations are performed in the regime ${\chi }_{1s} > {\chi }_{2s}$ using the parameters in panel (b).

**Extended Data Fig. 3:**
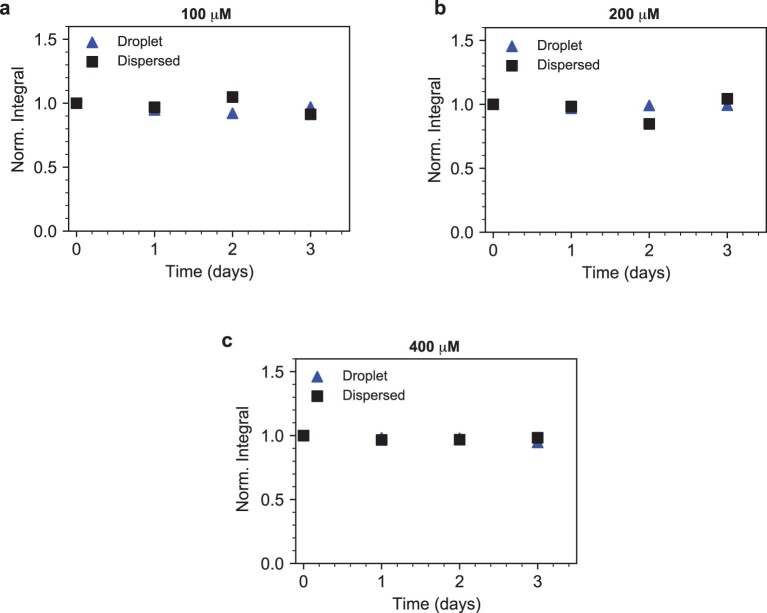
FUS fractions over time measured from the same protein stock at various concentrations. a, b, c) Both droplet and dispersed fractions of FUS did not change over the course of three days for all concentration measured (a: 100 μM, b: 200 μM, c: 400 μM).

To rationalize these observations, we developed a simple model that explores the relationship between FUS aggregation kinetics and phase separation using recently established theories for chemical reactions in coexisting phases at phase equilibrium^17,18^ (Extended Data Fig. 2a and Methods). We assume phase separation to be much faster than maturation. To understand the role of protein–solvent interactions, we varied the interaction parameters ${\chi }_{1{\mathrm{s}}},{\chi }_{2{\mathrm{s}}}$ between monomers ‘1’, aggregates ‘2’ and the solvent ‘s’. When monomer–solvent interactions are disfavored over aggregate–solvent interactions (${\chi }_{1{\mathrm{s}}} > {\chi }_{2{\mathrm{s}}}$), the solvent enters the liquid-droplet phase (Extended Data Fig. 2b), which could explain the increased diffusivity of the droplet fraction over time. By contrast, when ${\chi }_{1{\mathrm{s}}} < {\chi }_{2{\mathrm{s}}}$, the solvent is pushed out from the droplet phase because, in this case, the increasing maturant population disfavors solvent interactions (Extended Data Fig. 2c). Matured species are formed when protein–protein interactions are favored over solvent interactions. Therefore, the relevant regime is ${\chi }_{1{\mathrm{s}}} > {\chi }_{2{\mathrm{s}}}$, which supports our solution-state NMR data revealing a decrease in the one-dimensional (1D) NMR signal corresponding to the liquid-droplet phase. Interestingly, the model predicts that solvent flux into the droplet phase depends on the protein concentration (Extended Data Fig. 2d). Our results are consistent with a previous study showing how aggregation couples with changes in droplet volume over time^19^. Although these simulations show clear changes in solvent flux into the droplets that are consistent with our NMR data, other factors may also contribute to the observed changes in the NMR signal (Extended Data Fig. 3).

### FUS maturation is suppressed in the presence of RNA

Because FUS is known to play a role in RNA transcription^20–22^, we studied whether transcription could influence FUS maturation in vitro. To this end, we prepared in vitro transcription reactions as previously described^23^ and quantified the reaction velocities in the absence and presence of agarose-stabilized FUS droplets. Because no droplets are formed at 37 °C, the experiments were performed at 25 °C. Lowering the temperature resulted in slower reactions that reached saturation only after 2 days. This provided a sufficiently long time window to follow the velocity of transcription, and also the FUS droplet maturation process. Interestingly, we observed a strong, irreversible increase (up to 40%) in the condensed form of FUS (Fig. 1f) during the transcription reaction over 40 h. Without transcription, the droplet fraction of FUS is stable over time (Fig. 1f). As reported previously, low concentrations of RNA enhance droplet formation by RNA-binding proteins, whereas more RNA dissolves them^24^. Hence, observation of a large increase in the fraction of FUS in the condensed phase during the reaction indicates that the transcribed RNA interacts with FUS and further promotes its phase separation. Because the level of FUS in the droplet form is maintained for several days, this suggests that RNA promotes FUS liquid condensation over droplet maturation. Another interesting observation was made when comparing the solution-state NMR RNA imino signals during the time course of the reaction in the presence and absence of FUS. In the biphasic sample of FUS, we were able to measure a strong increase in the amount of RNA produced because of an increase in the initial transcription speed (Fig. 1g–i). Although further experiments are required to elucidate the cause of this effect, the increase in velocity could be attributed to crowding effects or interactions between FUS and reaction components that favor the polymerase kinetics.

### FUS maturation monitored by real-time solid-state NMR

To directly observe the maturation of liquid droplets and the formation of potential solid fibril species we turned to solid-state NMR, which can detect immobilized species and has previously been used successfully to determine the structure of the FUS fibril core (residues 39 to 95)^12^ and to study the maturation kinetics of a monophasic sample of FUS (1–163) in the condensed phase^14^ (Supplementary Table 1). Solid-state NMR has recently also been used to study the maturation of liquid droplets of short peptide-based condensates^25^.

**Fig. 2:**
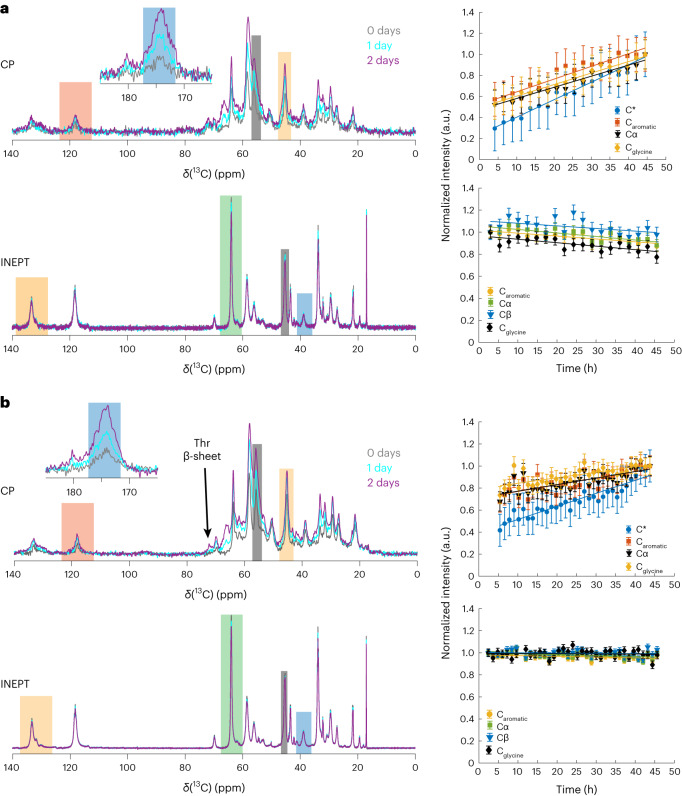
Maturation of FUS NTD liquid droplets followed by solid-state NMR in real time. **a**, Time-dependent ^1^H–^13^C CP-MAS and ^1^H–^13^C INEPT spectra of biphasic FUS (left) and time-dependent intensity changes during 2 days of maturation for several selected resonances (right; the spectral regions used are highlighted by colored rectangles in the spectra). The intensity of the CP spectrum recorded after 2 days was normalized to 1. A linear regression is shown (straight lines) with slopes (units of 1/days) of 0.398 (carbonyl carbons, C*), 0.290 (C_aromatic_), 0.254 (Cα) and 0.264 (Cα_glycine_) for the CP spectra, and −0.062 (C_aromatic_), −0.077 (Cβ), −0.058 (Cα) and −0.074 (Cα_glycine_) for the INEPT spectra. **b**, Time-dependent ^1^H–^13^C CP-MAS and ^1^H–^13^C INEPT spectra of monophasic FUS (left) and time-dependent intensity changes during 2 days of maturation for several selected resonances (right; the spectral regions used are highlighted by colored rectangles in the spectra). The intensity of the CP spectrum recorded after 2 days was normalized to 1. A linear regression is shown (straight lines) with slopes (units of 1/days) of 0.283 (C*), 0.144 (C_aromatic_), 0.142 (Cα) and 0.120 (Cα_glycine_) for the CP spectra, and −0.016 (C_aromatic_), −0.019 (Cβ), −0.006 (Cα) and −0.003 (Cα_glycine_) for the INEPT spectra. All normalized intensities data are presented as signal-to-noise values ± s.d.

**Extended Data Fig. 4:**
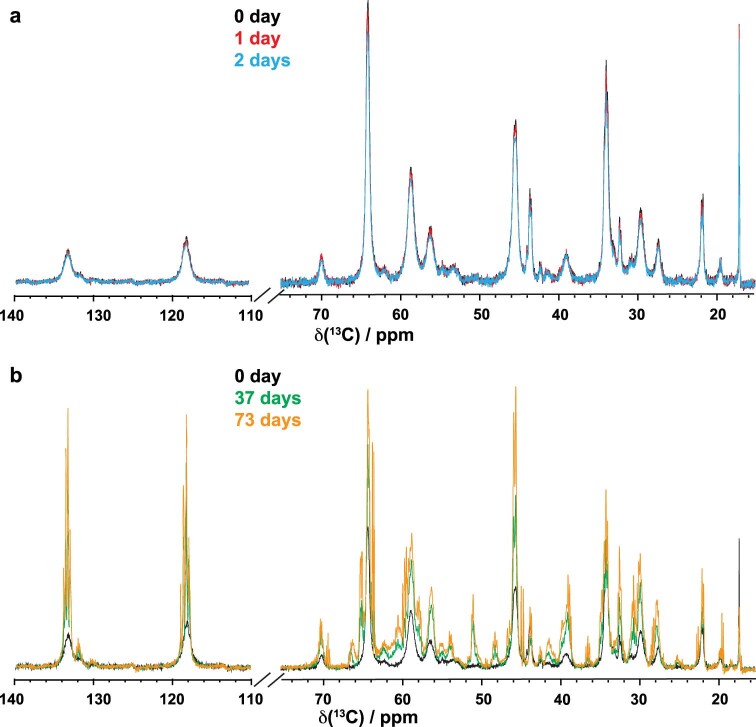
Comparison of 1D ^13^C-detected INEPT spectra for biphasic FUS-NTD liquid droplets recorded at various points in the maturation process. a) Almost no change in intensity is observed for the real-time INEPT signals up to two days of measurement after liquid droplet formation. b) Considerable increase in peak intensity, together with the appearance of *J*-resolved multiplet lines, can be appreciated already after 37 days. A further increase is observed after 73 days.

We tracked droplet maturation in our longer ^13^C,^15^N-labeled FUS NTD (1–267) sample in an agarose matrix (~8 mg of protein in the NMR rotor) over 2 days using real-time refocused insensitive nuclei enhancement by polarization transfer (INEPT) and cross-polarization (CP) NMR spectra, which allow the detection of soluble (highly mobile) and immobilized species, respectively^26–28^. Unlike the DOSY experiment, INEPT could not distinguish between NMR signals originating from the dilute and condensed phases. Nonetheless, we observed only a weak decrease in signal intensity (~10%) in the INEPT spectra over the first 2 days of maturation, followed by magic-angle spinning (MAS) (Fig. 2a and Extended Data Fig. 4a).

**Extended Data Fig. 5:**
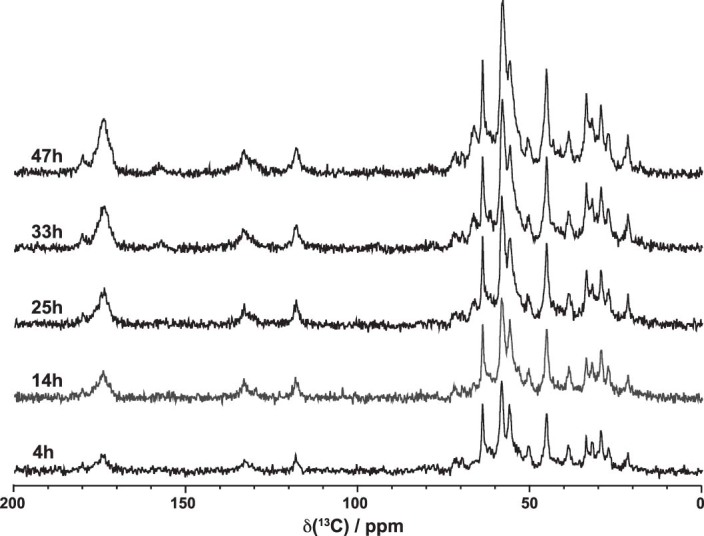
Overview of the time-dependent CP signal increase. In the first two days of maturation for 1D ^13^C-detected CP spectra of biphasic FUS-NTD recorded at different time points. Consistent signal increase together with the absence of chemical-shift perturbations can be observed, supporting the conclusion of solid aggregate formation with a similar conformation.

This agrees with our solution-state NMR observations, in which the net protein signals (the sum of the dilute and liquid-droplet phases) remained almost constant for the initial 3 days of maturation (Fig. 1c,e). By contrast, over a longer period (37 and 73 days), we detected an increase in the INEPT signal combined with a significant narrowing of the resonances caused most likely by an increase in the molecular-tumbling rate (Extended Data Fig. 4b). Turning to CP, we already detected a signal 4 h after rotor filling. The intensity of the CP signal increased over the course of 2 days, followed by a further increase within the next ~35 days, and no further changes after 73 days (Fig. 2a and Supplementary Fig. 1a,b). Note that comparison of the 1D CP spectra recorded in different measurement slots has an associated uncertainty in the peak intensity of roughly ±10%. We see no obvious chemical-shift perturbations during the first 2 days of maturation, indicating the gradual growth of a solid material that is already present 4 h after droplet formation (Extended Data Fig. 5). Quantitatively, half of the final CP signal observed after 2 days was already present after 4 h in the first CP spectrum (Fig. 2a), indicating that it is produced relatively quickly after droplet formation and spinning for the MAS experiment. Initial formation of solid material thus occurs very rapidly, and the subsequent increase is linear over time in this initial aging regime, but is not associated with a corresponding decrease in the INEPT signal as observed, for instance, in real-time solid-state NMR studies on protein aggregates^28^. This discrepancy between the INEPT-observed and CP-observed protein signal during FUS maturation has been previously reported and points to the potential presence of an NMR-invisible intermediate motional regime from which formation of the solid occurs^14^. Comparing the ^13^C CP spectra after 2 days of real-time MAS NMR and after 37 and 73 days revealed some spectral differences, indicating the formation of structurally distinct species after a longer maturation period (Supplementary Fig. 1a,b).

Moreover, visual inspection of the agarose-stabilized droplets after 6 months revealed the presence of suspended white particles. After extraction, we characterized the nature of these particles using electron microscopy and observed fibrils and fibril bundles (Supplementary Fig. 1c). Although further studies are required to identify the atomic details of these FUS fibrils, our NMR data indicate a different fibril fold compared with the shorter construct obtained upon seeding (1–163)^12^.

It is noteworthy that SDS–PAGE of fresh, 1-week-old and 1-month-old biphasic samples did not reveal any protein degradation (Supplementary Fig. 1d). Moreover, these were supernatants of biphasic samples without agarose (droplets were sedimented) thus probing the disperse phase. Similarly, visual inspection of the agarose-stabilized samples does not reveal any dissolvement of droplets. If indeed droplets were dissolving, the intensity of the gel bands would increase over time.

### Maturation rates in biphasic and monophasic samples

Another key difference from previous studies on FUS fibrils^14^ is our sample preparation protocol. In our study, FUS droplets were stabilized inside an agarose hydrogel matrix where they could mature as suspended droplets. In previous studies, droplets were centrifuged, resulting in a single bulk condensed phase from which the fibrils were formed and harvested. Liquid droplets possess a much larger combined surface compared with a bulk phase. To gain insight into the role of surfaces in maturation, we also tracked the development of solid-state NMR signals over time in a sample containing a single bulk condensed phase to compare it with a sample of liquid droplets. We used around 10 mg of protein sample in the 3.2-mm rotor for the bulk condensed phase (in the following this is denoted as the monophasic sample).

**Extended Data Fig. 6:**
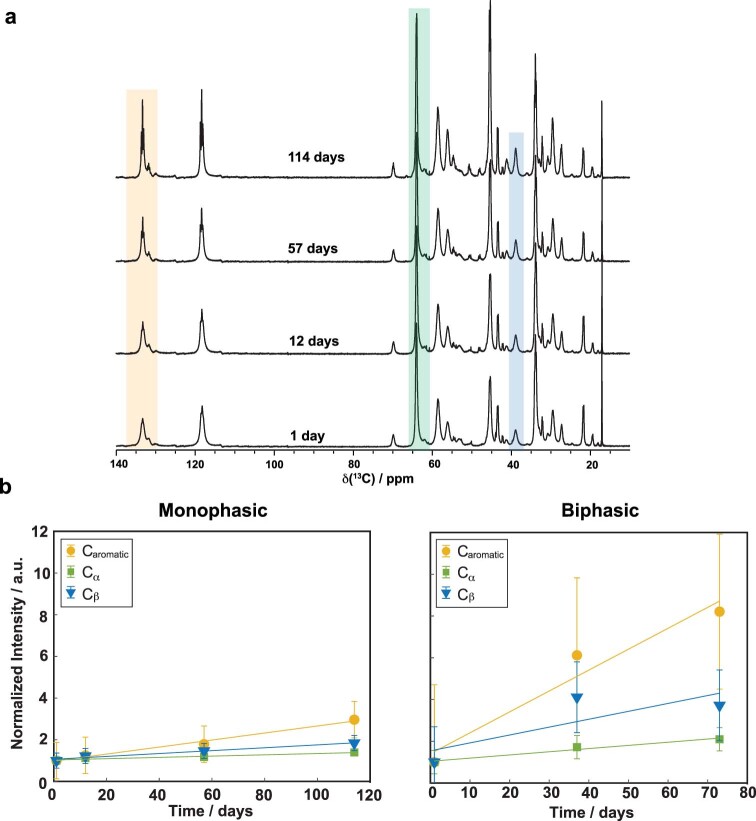
Overview of the signal increase in 1D ^13^C-detected INEPT spectra for monophasic FUS-NTD over 114 days of maturation. a) Stack plot of INEPT spectra recorded at four time points during the maturation period show an increase in signal intensity, accompanied by narrowing of the resonance lines). b) Comparison of the time dependence of the INEPT integrated intensities for the monophasic and biphasic sample indicates a smaller increase in intensity over time for the monophasic sample in comparison with the biphasic FUS-NTD. All normalized intensities data are presented as signal-to-noise values +/- standard deviation.

**Extended Data Fig. 7:**
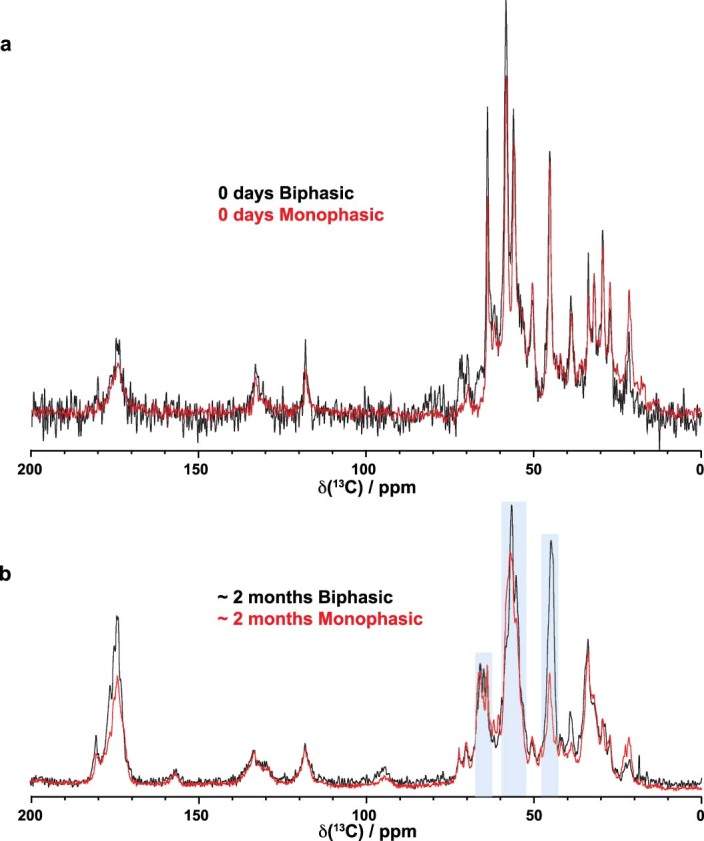
Comparison of 1D 13 C CP spectra of monophasic and biphasic FUS-NTD at the beginning of the maturation period (a) and after two months of storage at room temperature (b). a) The spectra look rather similar, albeit the threonine regions (~70 ppm) differ. b) After two months of maturation, notable differences can be observed, especially in the glycine region (~46 ppm) and carbonyl region for which a much more intense peak is observed for the biphasic sample. In a) the biphasic spectrum has been scaled up by a factor of 1.9 for ease of comparison.

**Extended Data Fig. 8:**
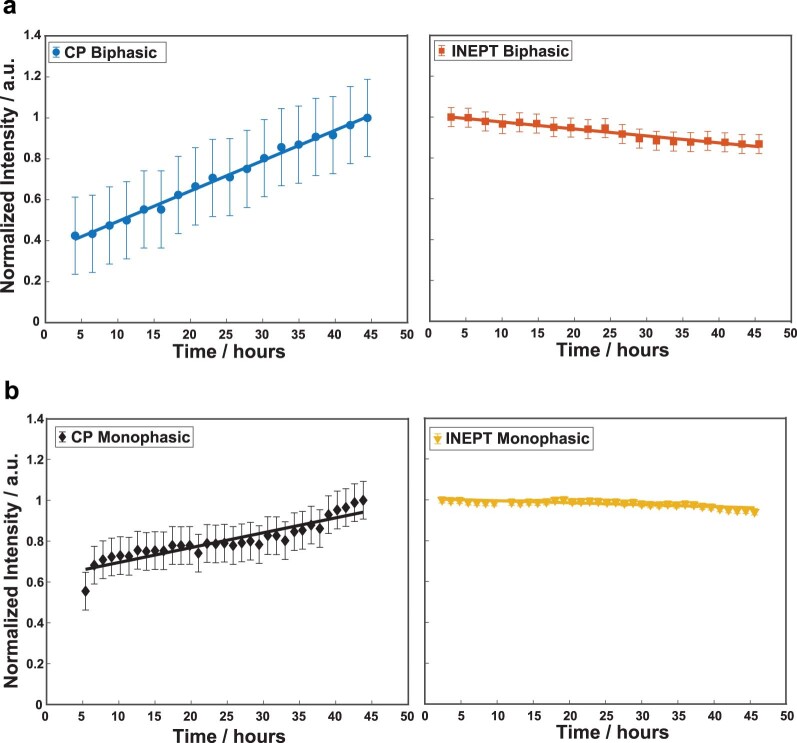
Maturation during the first two days. a) Time-dependent intensity changes for the absolute spectral integrals during two days of maturation for ^13^C,^1^H CP-MAS and ^13^C,^1^H INEPT spectra of biphasic FUS. The intensity of the total CP spectrum recorded after two days was normalized to one. A linear regression is shown (straight lines) with slopes of 0.346 a.u./days (CP), −0.082 a.u./days (INEPT). b) Time-dependent intensity changes for the absolute spectral integrals during two days of maturation for ^13^C,^1^H CP-MAS and ^13^C,^1^H INEPT spectra of monophasic FUS. The intensity of the total CP spectrum recorded after two days was normalized to one. A linear regression is shown (straight lines) with slopes of 0.214 a.u./days (CP), −0.022 a.u./days (INEPT). All normalized intensities data are presented as signal-to-noise values +/- standard deviation.

Similar to FUS droplets in the biphasic sample, the monophasic sample did not show any significant change in the INEPT signal intensities over 2 days (Fig. 2b). Over longer time scales, the relative intensity increase in the INEPT spectra was small compared with the biphasic sample (Extended Data Fig. 6a,b). Similar to the biphasic sample, some immobilized species were detected in the first CP measurement taken after 4 h (Fig. 2b). However, two decisive differences were observed in the maturation process between the two samples. The first is that at the beginning of the maturation period, the CP spectra are very similar (Extended Data Fig. 7a), but after 2 months of maturation they differ in peak positions and peak intensities (Extended Data Fig. 7b and the spectral regions highlighted therein). This is in line with the second major difference, namely changes in the maturation rate between the two samples. To obtain a rough comparison of the maturation rates, integrated resonances over time (normalized to the spectrum at 48 h for each case) were fitted linearly (see Supplementary Fig. 2 for the spectra of monophasic FUS NTD matured up to 114 days). Comparison of the slopes revealed that the biphasic sample, which contains even less protein than the monophasic sample, has a steeper slope, thus pointing to faster maturation than in the monophasic sample (Fig. 2a,b and Extended Data Fig. 8a,b). Despite this, the biphasic sample shows a clear linear increase in integrated CP resonances over time (Extended Data Fig. 8a); some deviations from this behavior are observed for the monophasic sample (Extended Data Fig. 8b).

Overall, the higher maturation rate found in the biphasic sample points to a clear role for the liquid-droplet surface. As mentioned previously^15^, the surface area-to-volume ratio is substantially larger in the biphasic sample, where droplets are stabilized, than in the monophasic bulk condensed phase, although under the high protein concentrations present in the NMR rotor partial clustering of liquid droplets cannot be ruled out. Because the two samples studied here also differ in agarose content, we investigated whether the droplet surface was the primary site for the maturation process.

### Structural differences between the surface and the interior

**Fig. 3:**
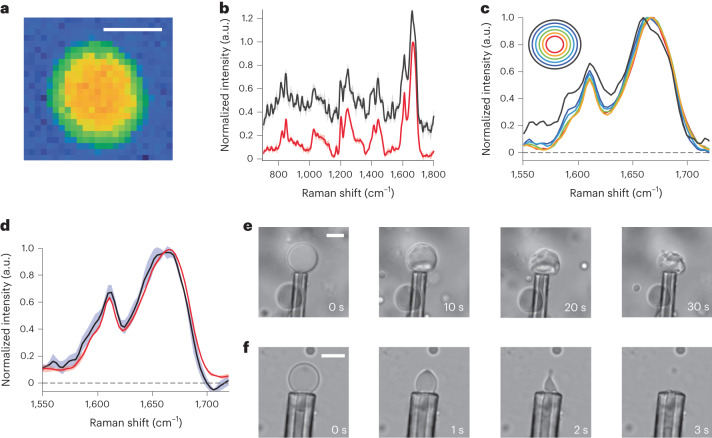
Characterization of FUS NTD droplet surface. **a**, Image of 2D scanned FUS NTD (residues 1–267) with the pixel contrast given by the integrated intensity of amide I band. Scale bar, 4 μm. **b**, Fingerprint region normalized averaged Raman-like spectra of 1-month-old droplets’ internal region (red) and border region (black). **c**, Comparison amide I band spectra of the concentrical rings regions of the droplets. The spectrum corresponding to the droplet surface is shown in black. **d**, Comparison amide I band spectra of the internal region (red) and border region (black) of fresh droplets. Shaded areas show the deviations between different samples. For **b** and **d** the mean and s.d. is shown from *n* = 5 different droplets, with more than 50 spectra averaged per droplet for border or center for each droplet. Shaded areas show the s.d. and the lines in the center are the means from each *n* = 5 droplets. **e**,**f**, Microaspiration of matured FUS droplets in the absence (**e**) and presence (**f**) of PolyU RNA measured in one experiment on three droplets each. The droplet can be completely aspirated only in presence of RNA, whereas in the absence of RNA a solid shell-like structure is revealed on the periphery of the droplet. Scale bar, 10 μm.

We used Raman spectroscopy to study structural differences at the surface of droplets. This method has the advantage of combining spatial resolution with protein secondary structure detection. By diluting FUS inside agarose hydrogel, micrometer-sized droplets were formed, and we measured the Raman spectra in distinct areas of 1-month-old droplets (Fig. 3a,b).

Analysis of the recorded spectra in the amide I region, which is sensitive to the protein secondary structure, revealed clear differences between the inside and the surface of the droplets. Further analysis of the spectra by segmenting droplets into concentric rings showed that the droplets possessed a core–shell-like structure: the interior of the droplets was largely homogeneous because the spectrum changed only very close to the droplet surface (Fig. 3c). Interestingly, normalized spectra revealed a more intense tyrosine peak at 1,618 cm^−1^ at the surface (Fig. 3c). Because this tyrosine peak is highly sensitive to hydrogen bonding, a more intense peak corresponds to tyrosines participating in a stronger hydrogen bond network, as expected from fibrils containing tyrosines in their cores^29^. The importance of tyrosine residues in liquid droplet maturation is further underlined by the rigidification of their side chains in the final fibrils suggesting π–π stacking, as shown by their appearance in the solid-state NMR CP spectra.

The spectrum at the surface of droplets exhibited a significantly narrowed peak at 1,665 cm^−1^ which is absent inside the droplet (Fig. 3c). Taken together, the spectra at the surface and inside the droplets clearly reveal spectral differences that point to significant structural changes occurring on the droplet surface upon maturation, whereas no changes are detected in fresh droplets (Fig. 3d).

Next, we tested the material properties by physically probing the droplets. Specifically, we aspirated material from a 1-month-old matured droplet to determine the material state and continuity. Although we could aspirate the liquid interior of the droplet, we observed that the periphery is made of a solid crust-like material that could not be aspirated and therefore collapsed (Fig. 3e and Supplementary Video 1). Interestingly, subsequent addition of poly-uridine (PolyU) RNA resulted in shell-less droplets that can be aspirated ten times faster at the same pressure and using similar sized droplets (Fig. 3f and Supplementary Video 2). Thus, it appears that the RNA can liquefy the already-rigid droplet shell, most likely by interacting nonspecifically with the RGG segment of the protein. This aligns with previous reports showing that RNA can preserve the liquid state of the FUS droplets and decrease the viscosity of the RGG-containing LAF-1 droplets^30,31^. This also agrees with our in vitro transcription experiment, in which RNA stabilizes FUS droplets and prevents their maturation (Fig. 1).

Because the bulk of aged droplets can be aspirated, we conclude that the bulk remains a liquid, confirming our solution-state NMR data and our theoretical model (Fig. 1 and Extended Data Fig. 2). Conversely, the surface appears solid, suggesting that it is a more structured material. The liquid–crust contrast between the bulk and the interface of the droplet in terms of aspiration agrees with the differences we see in the Raman spectra, and clearly points to a different FUS structure inside and at the surface of the droplet. In combination with solid-state NMR data, in which we observe structural changes during maturation leading to an increase in β-sheet content (vide infra), we propose that the crust-like material observed for mature droplets is likely to be primarily β-sheet in nature.

### FUS fibril secondary structure studied by solid-state NMR

**Fig. 4:**
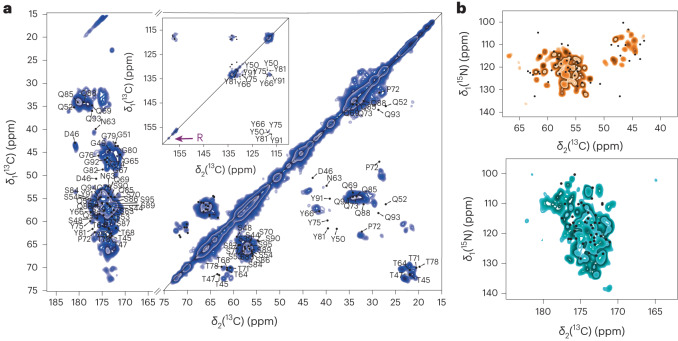
Two-dimensional solid-state NMR enables to characterize FUS NTD solid material. **a**, ^13^C–^13^C 20 ms DARR spectrum (left) of maturated monophasic FUS (68 days). The inset shows the aromatic region. A weak arginine side chain resonance is detected on the diagonal, highlighted by a purple arrow. The assigned peaks are back-predicted from Murray et al.^12^. **b**, ^15^N–^13^C NCA and NCO spectra of matured monophasic FUS (114 days). Dots represent peaks back-predicted from Murray et al.^12^. All spectra were recorded at 20.0 T and 17 kHz MAS.

To characterize the molecular structure of the solid material formed during the liquid-to-solid transition, we recorded two-dimensional (2D) ^13^C–^13^C dipolar assisted rotational resonance (DARR) spectra^32,33^ and ^15^N–^13^C NCA and NCO spectra of matured biphasic and monophasic samples^34^. Figure 4a shows the 2D DARR spectrum for the 68 days matured monophasic FUS sample. The spectrum is well-resolved with ^13^C line widths of ~1 ppm (~240 Hz), pointing to rather homogeneous fibrils formed during the phase-transition process. ^13^C Cα and Cβ chemical-shift values are sensitive reporters of the secondary structure^35^, which has been employed in structure calculations of amyloid fibrils^36^. For the spectrally well-resolved threonine and serine residues located in β-sheets, α-helices and loops, we plotted the statistical distribution of ^13^C Cα/Cβ chemical-shift values from probability density functions^37–39^ (Supplementary materials section and Supplementary Fig. 3). This analysis confirms the formation of β-sheets in matured samples, in agreement with previous studies of shorter FUS low complexity (LC) constructs (residues 1–163)^14^. Furthermore, we inspected the 1D ^13^C CP spectrum of the monophasic FUS NTD obtained after 2 days of maturation (Fig. 2b and Supplementary Fig. 3a,b) and observed the presence of threonine Cα/Cβ resonances characteristic of β-sheet secondary structures (~61.2 and 72.3 ppm for the Cα and Cβ resonances, respectively) showing that β-sheet appeared within the first 2 days of maturation. We thus plotted on the 1D ^13^C CP spectrum the threonine ^13^Cβ averaged chemical-shift values for the various secondary structure types (Supplementary Fig. 3b)^37^. Indeed, the high-frequency shifted threonine Cβ resonance falls within the β-sheet region. Further comparison of the 1D spectrum with the well-resolved threonine shifts in the 2D DARR of the sample after 68 days of maturation shows a clear match for the resonances (Supplementary Fig. 3c). Together with the Raman experiments, the fact that the biphasic and monophasic samples have almost identical 1D CP (Extended Data Fig. 7) allows us to propose that initial β-sheet formation takes place at the surface of liquid droplets and helps in formation of the crust observed during microaspiration.

**Extended Data Fig. 9:**
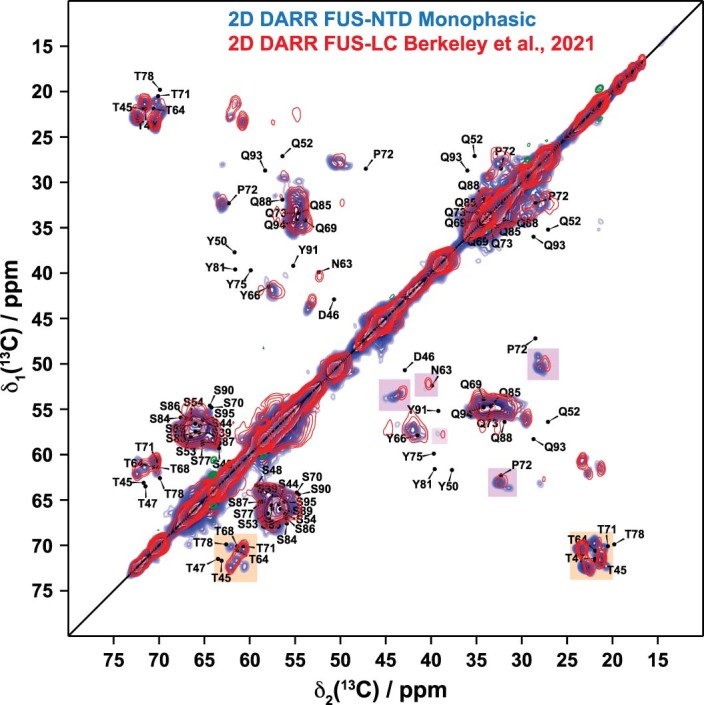
Comparison with previously published data. 2D DARR spectra for the maturated monophasic FUS-NTD in blue with the 2D DARR previously reported by^3^ in red. Black labels report on the back-predicted shifts for the FUS fibril core from^4^. In the figure, orange boxes represent additional resonances in our DARR spectrum, while purple squares indicate chemical-shift perturbations.

**Extended Data Fig. 10:**
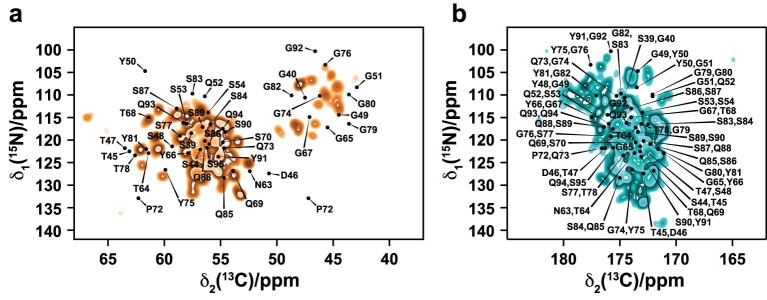
2D NCA (a) and NCO (b) spectra of matured monophasic FUS-NTD liquid droplets (sample stored at 114 days at r.t.). The assigned peaks are back-predicted from^4^. Both spectra were recorded at 20.0 T and 17 kHz MAS.

Comparing our DARR spectrum with one published previously on shorter FUS constructs reveals several differences (Extended Data Fig. 9)^14^. First, a variety of additional resonances is observed in our case (for instance, in the threonine region: orange boxes in Extended Data Fig. 9), together with noticeable chemical-shift perturbations for some well-isolated resonances (purple boxes in Extended Data Fig. 9). Second, we were also able to detect signals from arginine side chains in a ^15^N CP-MAS spectrum (Supplementary Fig. 4a), which apparently also rigidify to some extent after the phase-transition process. This is notable because arginine residues are present only in the RGG domain (absent in FUS LC), which suggests its engagement in the phase-separation process. The importance of the RGG domains in liquid–liquid phase separation (LLPS) has been reported recently in the context of the full-length protein^40^, and an indication of the role of the RGG domain in LLPS of FUS NTD is obtained from the solid-state NMR spectra presented herein. The matured FUS fibrils also show an intense CP signal for rigidified aromatic tyrosine side chains (Supplementary Fig. 3b,c), which are efficiently immobilized, most likely because of π–π stacking interactions or hydrogen bonds, as also indicated by the Raman spectra. Interestingly, and as reported previously^14^, the solid material formed via phase separation shows clear structural differences from FUS fibrils grown from fibril seeds (residues 1–214), for which the fibril core (residues 39–95) was structurally solved by solid-state NMR^12^. This is evident from the differences in chemical shifts between the LLPS-induced fibril state and the fibril core reported previously (see the back-predicted shifts for the FUS fibril core only plotted on the 2D DARR spectrum in Fig. 4a and the 2D NCA and NCO spectra in Fig. 4b and Extended Data Fig. 10, respectively). Similar conclusions can be drawn from the highly resolved heteronuclear NCA and NCO spectra shown in Fig. 4b. Because of the low amount of protein in the NMR rotor (~8 mg) caused by the preparation of liquid droplets, a sequential resonance assignment based on 3D spectra could not be performed and probably would require an ex situ matured sample. Unfortunately, because of the low signal-to-noise ratio (see Supplementary Fig. 5 for the 2D DARR spectrum), such an analysis was not possible with the current biphasic sample.

## Discussion

**Fig. 5:**
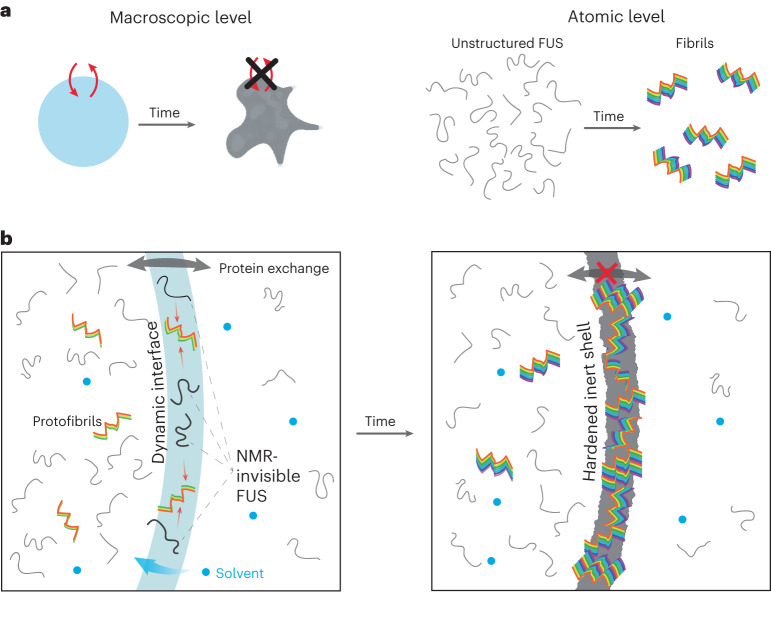
Current and proposed models for FUS maturation mechanism. **a**, Current view of maturation on the macroscopic and atomic levels. **b**, The proposed model. Fresh droplets have a highly dynamic interface with the solvent allowing protein exchange between phases. Although droplets are initially formed, a fraction of the protein instantaneously adopts a protofibrillar fold that is present both in the bulk of the droplets and on the surface. We propose the existence of a FUS population on the periphery, which because of the unique environment of the interface is not detected in conventional INEPT NMR experiments. Over time, this population is gradually incorporated into the fibrils of the surface. During the fibrilization process the hydrophobic nature of the droplet decreases as the hydrophobic groups of the protein are protected in the fibril core. This increases the flux of solvent molecules inside the droplet causing the diffusivity of the remaining monomers to increase. Once equilibrium is established, the whole surface is converted to fibrillar species resulting in the observed inert hardened shelled, which hinders material exchange between the phases.

Even though we are able to describe the maturation of liquid droplets both at the macroscopic (solidification) level and the atomic (fibrilization) level, we lack information on how these two aspects are connected and therefore on the mechanism behind the liquid-to-solid transition (Fig. 5a). Here, we propose a mechanism for the liquid-to-solid transition that incorporates our experimental observations (Fig. 5b).

Our data allowed us to better understand a previously reported NMR-invisible population of FUS^14^ that we detect with our solid-state INEPT decrease (Fig. 2a and Extended Data Fig. 4a). This spectroscopically invisible FUS may be positioned at the surface of droplets and be progressively converted to a solid crust where it becomes visible again (Fig. 5b). This state escapes detection by solid-state NMR because of its motion rate, which may cause inefficient heteronuclear polarization transfer by INEPT, as well as by CP experiments^41^. The role of the surface in maturation is supported by the faster rate of fibrilization of the biphasic sample compared with the monophasic sample as revealed by solid-state NMR, and the different structural composition detected on the surface of droplets compared with the droplet interior, as shown by Raman spectroscopy. The partial rigidification of arginine side chains in the RGG1 domain might indicate a different fibril core segment from that reported previously^12,42^, and this is also supported by significant chemical-shift differences between fibrils obtained from liquid droplet maturation and seeded fibrils. Alternatively, the partially rigidified arginine could correspond to surface-immobilized side chains, as proposed by the coarse-grained model of Garaizar and coworkers^43^. Subsequently, the concentration of fibrils increases on the surface, likely caused by a difference in surface tension^44^. During this process, water enters the droplets to balance the equilibrium between the two phases. The additional water decreases the viscosity inside the droplets; hence the DOSY NMR signal for the fast-diffusing FUS increases. Ultimately, this process results in a solid shell on the periphery that macroscopically indicates the passage from a liquid to a partially solid phase. This barrier between the dilute phase and the interior bulk droplet phase prevents the exchange of protein material, resulting in the observed limited recovery after photobleaching^45^.

The critical role of the droplet surface in liquid-to-solid transition is only starting to emerge because it was modeled recently^43,46^. Our work presents spectroscopic evidence that maturation occurs at the surface of liquid droplets. This is in agreement with previous reports of Thioflavin T accumulation and high Förster resonance energy transfer efficiency at the coacervates surface of TDP-43 and α-synuclein, respectively^47,48^. Thus it creates an additional cellular subcompartment to associate with molecular functions or exploit as a therapeutic target. Even though the concept of reactive liquid–liquid interfaces is not new in chemistry, where interfacial polymerization is utilized to obtain crystalline needles^49,50^, very few examples in biology have been reported. The synthesis of preribosomal RNA occurs at the interface between two of the three phases that comprise the nucleolus^51–53^. More recently, anisosomes were discovered in cells described as liquid spherical shells made of the RNA-binding protein TDP-43. Although both the droplet interior and the surrounding shell exhibited liquid properties, the latter was much more dense and functioned as a selective barrier^54^. This architecture of a dense shell resembles our macroscopic observation of a solid-like droplet periphery, and future studies should reveal whether it also shares the same function. Intriguingly, we report the that subcompartment of the droplet shell can be dissolved by RNA, which may indicate a specific function of the shell to orient charged side chains (arginines) as predicted^43^. Collectively, the inhomogeneity of the liquid droplets and the actual functional role of their surfaces indicate that the droplets are the means by which to form this important subcompartment, rather than the actual bulk condensed phase.

Maturation of FUS has been linked with disease evolution, although debate exists. FUS disease mutation G156E matures significantly faster than the wild-type^9^. If the matured species indeed causes the disease, then for a therapeutic approach it is mandatory not to perturb the stress granules but rather to prevent disease-relevant maturation. Therefore, instead of designing very specific molecules, we could repurpose already known molecules that partition into hydrophobic interfaces that act as wetting agents and ultimately interfere with maturation.

## Methods

### Protein expression and purification

FUS NTD (1–267) was expressed and purified under denaturing conditions as reported previously^15^. Double uniformly labeled protein (^15^N and ^13^C) for solid-state NMR experiments was expressed in M9 medium with ^15^N ammonium chloride and ^13^C glucose as the nitrogen and carbon source, respectively. Unless stated otherwise, the final stock concentration was 10 mM in 6 M urea buffer (50 mM HEPES, 150 mM NaCl, 6 M urea, pH 7.5).

### Solution-state NMR

Solution-state NMR samples were prepared as described previously^15^. Briefly, protein stock was diluted to the desired concentration using hot agarose buffer (30 mM HEPES, 200 mM KCl, 0.5% w/v agarose (Thermo Fisher Scientific), pH 7.3). The sample was transferred to a 3-mm NMR tube where the temperature gradually decreased to room temperature (25 °C), leading to agarose hydrogel and FUS liquid-droplet formation.

All solution-state NMR experiments were recorded at 25 °C using the following Bruker spectrometers with *z* axis pulsed field gradients: Avance III at 750 MHz proton frequency with a PATXI room-temperature probe (prep no. 1 and prep no. 5); Avance NEO at 500 MHz equipped with a CPQCI cryogenic probe (prep no. 2); Avance III HD at 600 MHz (prep no. 3); and Avance NEO at 700 MHz proton frequency equipped with a CP-TCI cryogenic probe. All spectra were processed with TopSpin 3.2 (Bruker Biospin).

A standard pulse sequence (stebpgp1s19 from TopSpin 3.2, Bruker Biospin) was used for diffusion experiments. In total, 4,096 points with 32 scans were recorded in the proton dimension for each dimension with variable diffusion gradient strength ranging between 2% and 95% in various steps. The following parameters were used: diffusion time (*Δ*) 0.05 s (except in Fig. 1c,d where *Δ* *=* 0.1 s), gradient pulse (*δ*) 10 ms, smoothed rectangular-shaped gradients SMSQ10.100 and relaxation delay (d1) 4 s.

### SDS–PAGE electrophoresis

FUS NTD protein stock was diluted in buffer (30 mM HEPES, 200 mM KCl, pH 7.3) to achieve a final concentration of 120 μM, resulting in a very turbid sample. The sample was then incubated at room temperature for up to 1 month. Samples (10 μl) to which SDS loading dye was added were taken 1 hour, 1 week and 1 month post preparation and kept at −20 °C. Once all samples had been collected, they were loaded onto a 15% acrylamide gel for electrophoresis. The gel was stained with Coomassie blue.

### In vitro transcription reactions

The in vitro transcription reactions were designed according to the previously published protocol^23^ with an additional 0.5% w/v agarose, if stated. The RNA transcribed is an intronic splicing regulator (downstream control sequence) from the c-*src* gene^55^. Template DNA is cloned in a PTX1 (ref. ^56^) vector linearized overnight at 37 °C with BsaI enzyme. The transcription reaction buffer (40 mM Tris–HCl, pH 7.7, 0.01% Triton X-100, 5 mM DTT) was supplemented with 5 mM from each NTP, 24 mM MgCl_2_, 1 U ml^−1^ inorganic pyrophosphatase from baker’s yeast, 200 μM FUS NTD (if added) and 0.3 μM T7 RNA polymerase. Finally, highly concentrated agarose (1.5% w/v) was added to achieve a 0.5% final concentration and was transferred quickly, while liquid, to a 3-mm NMR tube using a long glass pipette.

### Theoretical model of FUS maturation in coexisting phases

We devised a simple model of FUS maturation in coexisting phases that accounts for protein–solvent interactions, following previous studies^17,18^. The model considers an incompressible, ternary mixture of monomers, aggregates and solvent with volume fractions ${\phi }_{1}$, ${\phi }_{2}$ and ${\phi }_{s}$ respectively. Monomers and aggregates can undergo phase separation as described by the Flory–Huggins free-energy density, *f* (refs. ^57–59^),

1 $$\begin{array}{l}\frac{f}{{k}_{\mathrm{B}}T}=\frac{{\phi }_{1}}{{v}_{1}}\mathrm{ln}\left({\phi }_{1}\right)+\frac{{\phi }_{2}}{{v}_{2}}\mathrm{ln}\left({\phi }_{2}\right)+\frac{{\phi }_{\mathrm{s}}}{{v}_{\mathrm{s}}}\mathrm{ln}\left({\phi }_{\mathrm{s}}\right)\\\qquad+{\chi }_{12}{\phi }_{1}{\phi }_{2}+\left(\;{\chi }_{1\mathrm{s}}{\phi }_{1}+{\chi }_{2\mathrm{s}}{\phi }_{2}\right){\phi }_{\mathrm{s}},\end{array}$$

where *k*_B_*T* denotes thermal energy, ${v}_{i}$ is the molecular volume of component $i$ (*i* = 1,2,s) and ${\chi }_{{ij}}$ are the Flory–Huggins interaction parameters describing the effective interaction between components *i* and $j$ (monomer–solvent, aggregates–solvent, monomer–aggregates). Phase equilibrium between the droplet (I) and dilute (II) phases results from solving the phase-coexistence conditions:

2 $${\mu }_{1}^{{\mathrm{I}}}={\mu }_{1}^{{{\mathrm{II}}}},{\mu }_{2}^{{\mathrm{I}}}={\mu }_{2}^{{{\mathrm{II}}}},{\varPi }^{{\mathrm{I}}}={\varPi }^{{{\mathrm{II}}}},$$

where ${\mu }_{i}={v}_{\mathrm{s}}\frac{\partial f}{\partial {\phi }_{i}}$ is the chemical potential of component *i*, $\varPi =-f+{\phi }_{1}\frac{\partial f}{\partial {\phi }_{1}}+{\phi }_{2}\frac{\partial f}{\partial {\phi }_{2}}$ is the osmotic pressure, and the superscript I/II indicates the dense/dilute phase, respectively.

In addition to undergoing phase separation, the monomers can aggregate. In each phase, we describe this process as a transition between monomeric and aggregated states via the simple reaction scheme ${\phi }_{1}\rightleftharpoons {\phi }_{2}$. We note that the model can, in principle, be generalized to account for more complex aggregation events, including primary nucleation, elongation, fragmentation or secondary nucleation^60^, but further studies are required to understand the relative contribution of these aggregation steps to the overall aggregation process in droplets. We assume that the solvent is nonreactive and that the forward (${k}_{1}$) and backward (${k}_{2}$) rates are phase independent (they are identical in the dense and dilute phases). Under these conditions, we obtain the following kinetic equation for the average volume fractions of monomers and aggregates, ${\overline{{\phi }}_{i}}=({V}^{\;{\mathrm{I}}}/V\;){\phi }_{i}^{{\mathrm{I}}}+\left({V}^{{\;{\mathrm{II}}}}/V\right){\phi }_{i}^{{{\mathrm{II}}}}$ (ref. ^17^):

3 $$\frac{{\mathrm{d}}{\overline{{\phi }}_{1}}}{{{\mathrm{d}}t}}=-{k}_{1}{\overline{{\phi }}_{1}}+{k}_{2}{\overline{{\phi }}_{2}}=-\frac{{\mathrm{d}}{\overline{{\phi }}_{2}}}{{{\mathrm{d}}t}}.$$

We consider a situation in which phase separation is much faster than aggregation. Under these conditions, phase equilibrium is established almost instantly during aggregation. Therefore, we can solve the aggregation kinetics, equation (3), for the average monomer and aggregate volume fractions starting from a monomeric solution ${\overline{{\phi }}_{1}}\left(0\right)={\phi }_{{{\mathrm{tot}}}}$

4a $${\overline{{\phi }}_{1}}\left(t\right)=\frac{{\phi }_{{{\mathrm{tot}}}}}{{k}_{1}+{k}_{2}}\left({k}_{2}+{k}_{1}{\mathrm{e}}^{-\left({k}_{1}+{k}_{2}\right)t}\right)$$

4b $${\overline{{\phi }}_{2}}\left(t\right)=\frac{{\phi }_{{{\mathrm{tot}}}}}{{k}_{1}+{k}_{2}}\left({k}_{1}-{k}_{1}{\mathrm{e}}^{-\left({k}_{1}+{k}_{2}\right)t}\right)$$

and at every time point during aggregation calculate the phase-separation equilibrium of the resulting monomer/aggregate mixture using phase-coexistence conditions (equation (4)). This construction allows us to follow the time evolution of the monomer, aggregate and solvent concentrations in the dense (I) and dilute (II) phases, yielding the plots in Extended Data Fig. 2.

To generate the plots, we used the following parameters: ${v}_{1}={v}_{2}={v}_{\rm{s}}$, ${\chi }_{12}=0$, ${{\phi }_{1}\left(0\right)=\phi }_{{{\mathrm{tot}}}}=0.55$, ${\phi }_{2}\left(0\right)=0$, ${\chi }_{1\mathrm{s}}=3$, ${\chi }_{2\mathrm{s}}=2.1,$ ${k}_{1}=0.2\;{{{\mathrm{d}}}}^{-1}$, ${k}_{2}=0.1\;{{{\mathrm{d}}}}^{-1}$ (Extended Data Fig. 2b) and ${v}_{1}={v}_{2}={v}_{\mathrm{s}}$, ${\chi }_{12}=0$, ${{\phi }_{1}\left(0\right)=\phi }_{{{\mathrm{tot}}}}=0.55$, ${\phi }_{2}\left(0\right)=0$, ${\chi }_{1\mathrm{s}}=2.1$, ${\chi }_{2\mathrm{s}}=3,$ ${k}_{1}=0.1\;{{{\mathrm{d}}}}^{-1}$, ${k}_{2}=0.05\;{{{\mathrm{d}}}}^{-1}$ (Extended Data Fig. 2c). For Extended Data Fig. 2d the parameters are: ${v}_{1}={v}_{2}={v}_{\mathrm{s}}$, ${\chi }_{12}=0$, ${\phi }_{2}\left(0\right)=0$, ${\chi }_{1\mathrm{s}}=3$, ${\chi }_{2\mathrm{s}}=2.1$ with different initial conditions ${{\phi }_{1}\left(0\right)=\phi }_{{{\mathrm{tot}}}}=0.55$ (solid lines) and ${{\phi }_{1}\left(0\right)=\phi }_{{{\mathrm{tot}}}}=0.8$ (dashed lines).

### Microaspiration

The protein stock concentration for this experiment was 2 mM. The droplets were prepared by dilution to a final concentration of 100 μM using buffer without agarose (30 mM HEPES, 200 mM KCl, pH 7.3) and were matured at room temperature over a period of 1 month. For this experiment, a micropipette with a 5-μm tip was treated with BSA both on the inside and outside to prevent any clogging or adhesion of the condensed phase to the glass. PolyU RNA (Sigma Aldrich) was solubilized in the above buffer at a stock concentration of 2 mg ml^−1^. PolyU was added at a final concentration of 0.1 mg ml^−1^. An external pump controlled the applied pressure of the micropipette (Δ*P* = 820 Pa) and a bright field microscope was used to visualize the microaspiration.

### Electron microscopy

A FUS NTD biphasic sample was matured for 6 months at room temperature inside a 3-mm NMR tube sealed using nail polish. The agarose hydrogel was extracted from the NMR tube by breaking the bottom of the tube. A 2-mm piece of hydrogel was cut into multiple small pieces and 50 μl of dilution buffer (30 mM HEPES, 200 mM KCl, pH 7.3) was subsequently added. The sample was sonicated for 30 min at room temperature.

Continuous carbon-supported copper grids (Quantifoil, Cu 300) were glow discharged (PELCO easiGlow, Ted Pella, negative, 25 mA, 30 s). After that, 3 µl of the sample was applied to the grids and incubated for 1 min at room temperature. The grids were then blot dried, washed with two drops of distilled water, and stained with two drops of 1% uranyl acetate for 30 s. Micrographs were acquired using a Tecnai F20 (Thermo Fisher Scientific) microscope operated at 200 kV and equipped with a Falcon II camera at ×62,000 magnification (pixel size: 1.7 Å per pixel) at a total dose of approximately 50*e*^−^ using EPU (v.1.6.0) software (Thermo Fisher Scientific). The targeted defocus for the data acquisition was set to −3 µm.

### Preparation of stabilized FUS droplets for coherent anti-Stokes Raman scattering imaging

An agarose solution (0.35% w/v) of ultra-low melt agarose (Sigma) in dilution buffer (30 mM HEPES, 200 mM KCl, pH 7.3) was made in a 15-ml conical tube. The tube was then placed in a Thermomixer (Eppendorf) operating at 90 °C, with shaking at 300 rpm until the solution was clear. This stock solution was used within 2 days.

To make samples, we used Grace Bio-Labs SecureSeal Imaging spacers as gaskets on standard glass slides. The solution of agarose was placed in the Thermomixer and heated to 90 °C at 300 rpm for at least 10 min to ensure it was warm. The gasket’s protective films were removed and the gasket was stuck to a glass slide. Then 18 µl of warmed agarose solution was placed into the free space in the center of the gasket, and 2 µl of FUS stock (1.2 mM in storage buffer) was immediately added to this solution and very gently pipetted. Droplet formation was noted by initially observing a whitish ring that diffused radially outward after the addition of the FUS stock to the agarose solution. A coverslip was then placed on top of the gasket, the sides of the coverslip were pressed down and the sample was sealed with nail polish. These samples were stable for more than 3 months, judging by the lack of obvious evaporation in the sealed sample.

As-prepared samples were allowed to sit for 2 h before measuring, and are termed ‘fresh’ samples for the coherent anti-Stokes Raman scattering (CARS) studies in this work. Samples were measured, then stored in a drawer in the laboratory until the next measurement; for example, 1 month later.

### CARS imaging of stabilized FUS droplets

For the CARS measurements, samples were removed from the cabinet drawer and placed on the microscope. Our broadband coherent anti-Stokes Raman scattering (BCARS) microscope has been described elsewhere, and its application for liquid droplets has also been previously presented^61,62^. Briefly, the pump/probe and Stokes pulses are generated in a dual-output, sub-nanosecond laser source (CARS-SM-30, Leukos), spatially and temporally overlapped at the sample plane of an inverted microscope (Eclipse Ti-U, Nikon), and tightly focused on the sample using a ×100, 0.85 numerical aperture air objective (LCPlan N, Olympus). The BCARS signal is filtered from the excitation pulses and focused onto the slit of a spectrograph (Shamrock 303i, Andor), which disperses the spectral components on a cooled charge-coupled device camera (Newport DU920P-BR-DD, Andor). Samples were mounted with the cover slip facing the objective. The samples were then raster scanned by moving a piezo stage (Nano-PDQ 375 HS, Mad City Labs), and the data acquisition was controlled via interface software in LabView 2015 (National Instruments).

Collected hyperspectral data were processed afterward in IgorPro (Wavemetrics) to extract the Raman-like spectra. The Raman-like spectra were obtained by phase-retrieval via a modified Kramers–Kronig transform using the surrounding agarose solution as the nonresonant^63^. The remaining error phase was removed using a Savitzky–Golay filter with a second-order polynomial and window size of 400 cm^−1^, producing the Raman-like spectra for this work.

### Solid-state NMR spectroscopy

FUS NTD uniformly ^13^C-labeled and ^15^N-labeled protein was mixed in 1:10 ratio with dilution buffer without agarose (30 mM HEPES, 200 mM KCl, pH 7.3) to form droplets. For the biphasic samples, the droplets were centrifuged (25 °C, 10,000*g*, 10 min), resulting in sedimented droplets approximately 30 μl in size and the top dilute phase was removed. The droplet phase was resuspended in 50 μl of agarose containing dilution buffer and transferred quickly to the NMR rotor. For the monophasic samples, droplets were centrifuged and sedimented already in the rotor forming the single condensed phase.

Solid-state NMR spectra were recorded at 20.0 T static magnetic-field strength in a 3.2-mm Bruker ‘Efree’ probe^64^. The MAS frequency for all the experiments was set to 17 kHz. All spectra were processed with the software TopSpin (v.4.1.3, Bruker Biospin). The 2D spectra were processed with a shifted (DARR monophasic: 2.5, NCA/NCO monophasic: 3, DARR biphasic: 2) squared cosine apodization function and automated baseline correction in the indirect and direct dimensions. The sample temperature was set to 278 K^65^. All spectra were analyzed with the software CcpNmr (v.2.4.2) and referenced to 2,2-dimethyl-2-silapentane-5-sulfonate^66,67^. The experimental parameters used are summarized in Supplementary Table 2.

### Analysis of real-time solid-state NMR kinetics

Kinetic analysis of the time-dependent intensities from the 1D spectra of biphasic and monophasic FUS was carried out by manually extracting individual signal-to-noise values of some representative peaks (Fig. 2a,b), as well as absolute integral values (Extended Data Fig. 8), of each spectrum in the time-dependent series via the build-in TopSpin module SiNo (signal-to-noise calculator, intensity of a peak divided by the square of the noise intensity). The intensities of interest were then loaded, visualized and processed in MATLAB (v.R2021b, MathWorks).

### Secondary structure chemical-shift predictions

Average secondary structure-dependent chemical-shift values for threonines and serines and their associated standard deviations were taken from Wang and Jardetzky^37^ and visualized on the 1D CP spectrum using a home-written MATLAB script (v.R2021b, MathWorks). The 2D probability density distribution plots for the secondary structure chemical-shift statistics were estimated and visualized using the PLUQin (https://github.com/kfritzsc/pluq) python package, for which the raw data were extracted from the PASCY/BMRB database^38,39^.

### Reporting summary

Further information on research design is available in the Nature Portfolio Reporting Summary linked to this article.

## Online content

Any methods, additional references, Nature Portfolio reporting summaries, source data, extended data, supplementary information, acknowledgements, peer review information; details of author contributions and competing interests; and statements of data and code availability are available at 10.1038/s41589-024-01573-w.

### Supplementary information

Supplementary Information Supplementary Tables 1 and 2, and Figs. 1–5.

Reporting Summary

Supplementary Video 1 Microaspiration of FUS liquid droplets.

Supplementary Video 2 Microaspiration of FUS liquid droplets in presence of RNA.

## Data Availability

Data supporting the findings of this study are available within the paper, its Supplementary Information and publicly available database (10.5281/zenodo.10708805).
