# Supplementary material for: A solid beta-sheet structure is formed at the surface of FUS droplets during aging
Source: Nat Chem Biol. 2024 Mar 11;20(8):1044–52. doi: 10.1038/s41589-024-01573-w (PMC11288893; doi:10.1038/s41589-024-01573-w)
Supplement: Supplementary file 1 — Supplementary Tables 1 and 2, and Figs. 1–5. [file 41589_2024_1573_MOESM1_ESM.pdf]

nature chemical biology

Article

https://doi.org/10.1038/s41589-024-01573-w

A solid beta-sheet structure is formed at the surface of FUS droplets during aging

In the format provided by the authors and unedited

<u>Supplementary Material:</u>

A solid beta-sheet structure is formed at the surface of FUS droplets during aging

Supplementary Table 1: Overview of solid-state NMR investigations performed on different FUS constructs.

FUS sequence	Solid-state NMR investigations	Reference
1-214<br>monophasic	Structure determination of the fibril core (39-95)	Murray et al., 2017
1-163<br>monophasic	Maturation kinetics, structural studies	Berkeley et al., 2021
1-267<br>mono- and biphasic	Maturation kinetics, structural studies	This work

Supplementary Table 2: Overview about experimental parameters of the performed solid-state NMR experiments. For more details about the used adiabatic CP steps and the tangential shapes used see[1].

**Sample**	**FUS NTD (monophasic)**	**FUS NTD (monophasic)**	**FUS NTD (monophasic)**	**FUS NTD (monophasic)**	**FUS NTD (monophasic)**	**FUS NTD (monophasic)**
**Experiment**	**1D ^{13}C CP**	**1D ^{13}C INEPT**	**2D DARR 20 ms**	**1D ^{15}N,^{1}H CP-MAS**	**2D NCA**	**2D NCO**
ν_r / kHz	17	17	17	17	17	17
B_0 / T	20	20	20	20	20	20
**Transfer I**	**HC-CP**	**HC-INEPT**	**HC-CP**	**HN-CP**	**HN-CP**	**HN-CP**
$\nu_1(^1H)$ / kHz	60	-	60	60	60	60
$\nu_1(X)$ / kHz	41.6	-	38	47	-	-
$\nu_1(Y)$ / kHz	-	-	-	-	43	43
Shape	Tangent ^{1}H	-	Tangent ^{1}H	Tangent ^{1}H	Tangent ^{1}H	Tangent ^{1}H
^{13}C carrier / ppm	100	100	100	-	-	-
^{15}N carrier / ppm	-	-	-	120	120	120
CP contact time / ms	0.6	-	0.6	1.2	1.2	1.2
**Transfer II**	-	-	**DARR**	-	**NC-CP**	**NC-CP**
$\nu_1(^1H)$ / kHz	-	-	17	-	-	-
$\nu_1(X)$ / kHz	-	-	-	-	6	6
$\nu_1(Y)$ / kHz	-	-	-	-	11	11
^{13}C carrier/ ppm	-	-	100	-	100	100
CP contact time / ms	-	-	20	-	6.5	6.0
t_1 increments	3072	16384	1536	3072	3072	3072
Sweep width (t_1) / ppm	100	100	100	100	117	117
Acquisition time (t_1) / ms	15.4	81.9	7.7	15.4	15.4	15.4
t_2 increments	-	-	3’072	-	192	192
Sweep width (t_2) / ppm	-	-	100	-	16	16
Acquisition time (t_2) / ms	-	-	15.4	-	0.7	0.7
^{1}H Spinal-64^a or WALTZ-64^b decoupling / kHz	90^a	5^b	90^a	90^a	90^a	90^a
Interscan delay / s	2	2	2.7	1.5	2.7	2.7
Number of scans	1024	1024	36	1024	112	160
Measurement time / h	0.5	0.5	42	0.4	16.5	23.5

Supplementary Table 2 continued.

**Sample**	**FUS NTD (biphasic)**	**FUS NTD (biphasic)**	**FUS NTD (biphasic)**
**Experiment**	**1D ^{13}C CP**	**1D ^{13}C INEPT**	**2D DARR 20 ms**
ν_r / kHz	17	17	17
B_0 / T	20	20	20
**Transfer I**	**HC-CP**	**HC-INEPT**	**HC-CP**
$\nu_1(^1H)$ / kHz	60	-	60
$\nu_1(X)$ / kHz	40.8	-	40.8
Shape	Tangent ^{1}H	-	Tangent ^{1}H
^{13}C carrier / ppm	100	100	100
CP contact time / ms	0.6	-	0.6
**Transfer II**	-	-	**DARR**
$\nu_1(^1H)$ / kHz	-	-	17
^{13}C carrier/ ppm	-	-	100
CP contact time / ms	-	-	20
*t*1 increments	3072	16384	2560
Sweep width (*t*1) / ppm	100	100	100
Acquisition time (*t*1) / ms	15.4	81.9	12.8
*t*2 increments	-	-	3'072
Sweep width (*t*2) / ppm	-	-	100
Acquisition time (*t*2) / ms	-	-	15.4
^{1}H Spinal-64[a] or WALTZ-64[b] decoupling / kHz	90[a]	5[b]	90[a]
Interscan delay / s	2	2	2.5
Number of scans	2048	2048	24
Measurement time / h	1.1	1.1	43

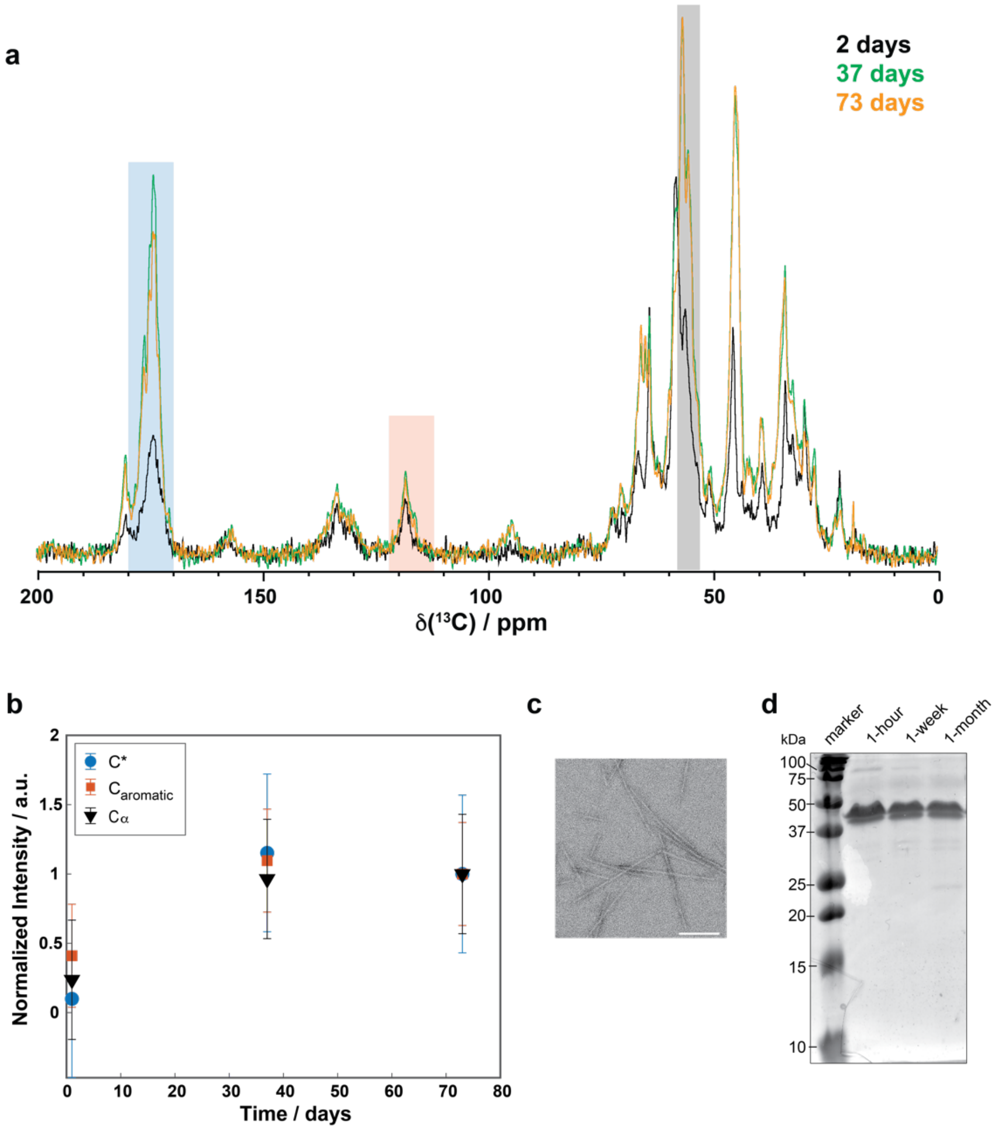

Supplementary Fig. 1: Maturation of biphasic FUS NTD. a) Comparison of 1D ^{13}C-detected CP spectra of biphasic FUS-NTD recorded at various points in the maturation process. The signal intensity stays rather constant over long-term maturation (i.e. from 37 to 73 days), indicating a plateau in the fibrilization process. b) The plateau in fibrilization is also confirmed by the kinetic analysis of the integrated intensities, which show small changes in intensity (within standard deviation) after 37 days and 73 days of storage. All normalized intensities data are presented as means of signal-to-noise values +/- standard deviation. c) Electron-microscopy image of FUS fibrils taken on a biphasic sample matured for six months. Scale bar : 100 nm. d) Different time points from biphasic sample in a 15% SDS-PAGE gel. These samples do not contain agarose to allow electrophoresis, therefore already after 1-hour from sample preparation droplets have sedimented. Protein concentration is 120 μM. Note, despite the molecular weight of the protein (26 kDa), it appears routinely at approximately 40 kDa. Both c and d performed twice with similar results.

a

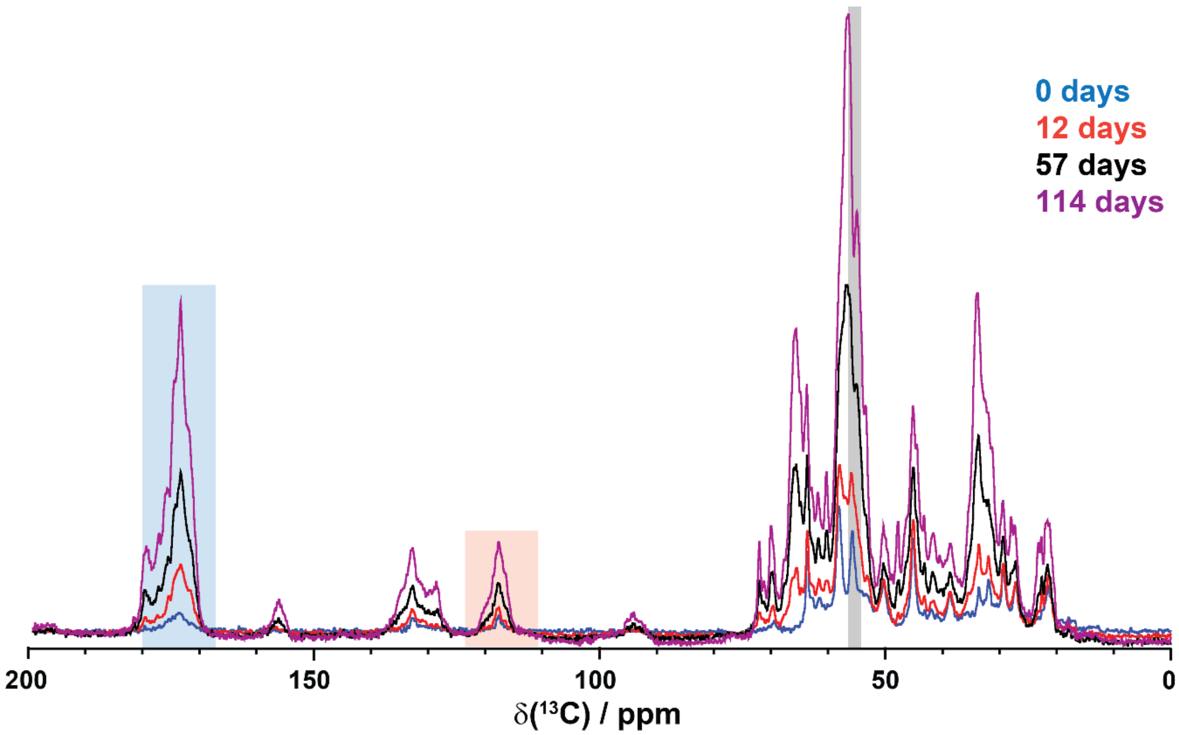

b

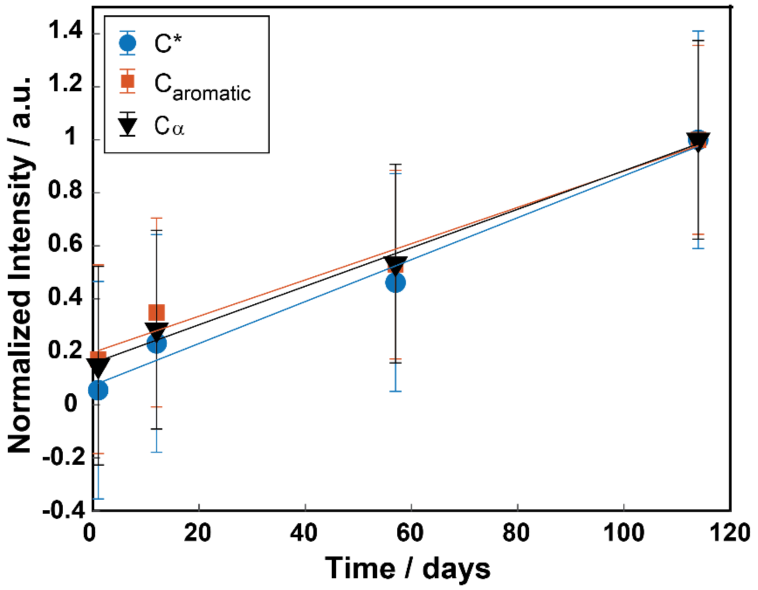

Supplementary Fig. 2: Maturation of monophasic FUS NTD. a) Comparison of 1D ^{13}C-detected CP spectra of monophasic FUS-NTD recorded at various points in the maturation process. Differently from the biphasic sample, the signal intensity increases consistently over the whole time up to 114 days. b) The kinetic analysis of the integrated intensities shows a rather linear increase over time. Spectral regions highlighted in a) were used for the analysis. All normalized intensities data are presented as means of signal-to-noise values +/- standard deviation.

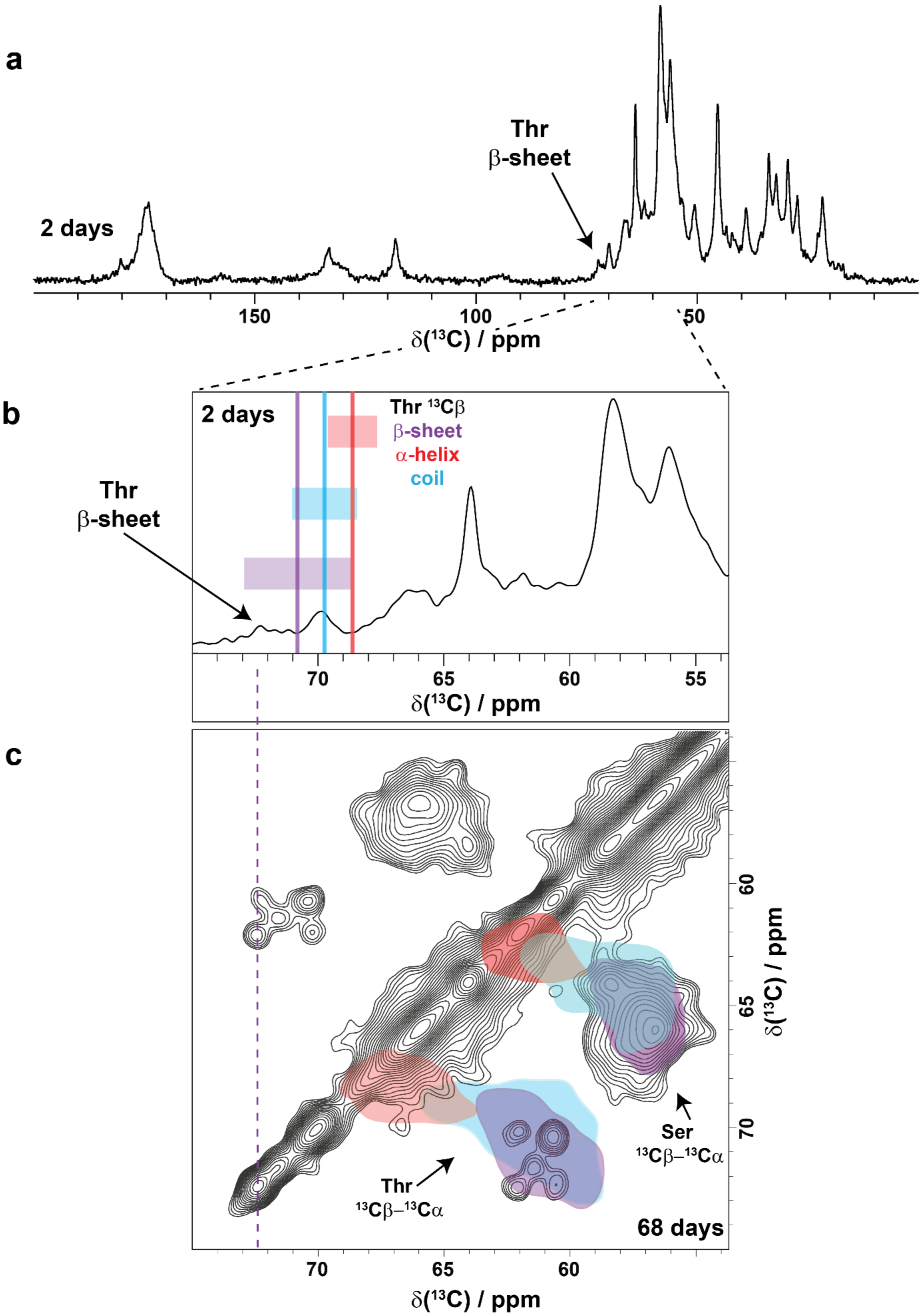

Supplementary Fig. 3: Secondary-structure chemical shift statistics indicates β-sheet formation. a) 1D ^{13}C CP spectrum of monophasic FUS NTD recorded 2 days after preparation. b) Zoom into the ^{13}C Cα/Cβ region of the spectrum with threonine Cβ chemical shift statistics plotted on top. c) Zoom into the 2D ^{13}C-^{13}C DARR spectrum of monophasic maturated (68 days) FUS-NTD together with chemical-shift predictions for the well-resolved threonine and serine resonances. The details for the secondary-structure predictions of ^{13}C Cα and Cβ chemical-shift values are reported in the material and methods section. The three types of secondary structure elements are highlighted with different colors.

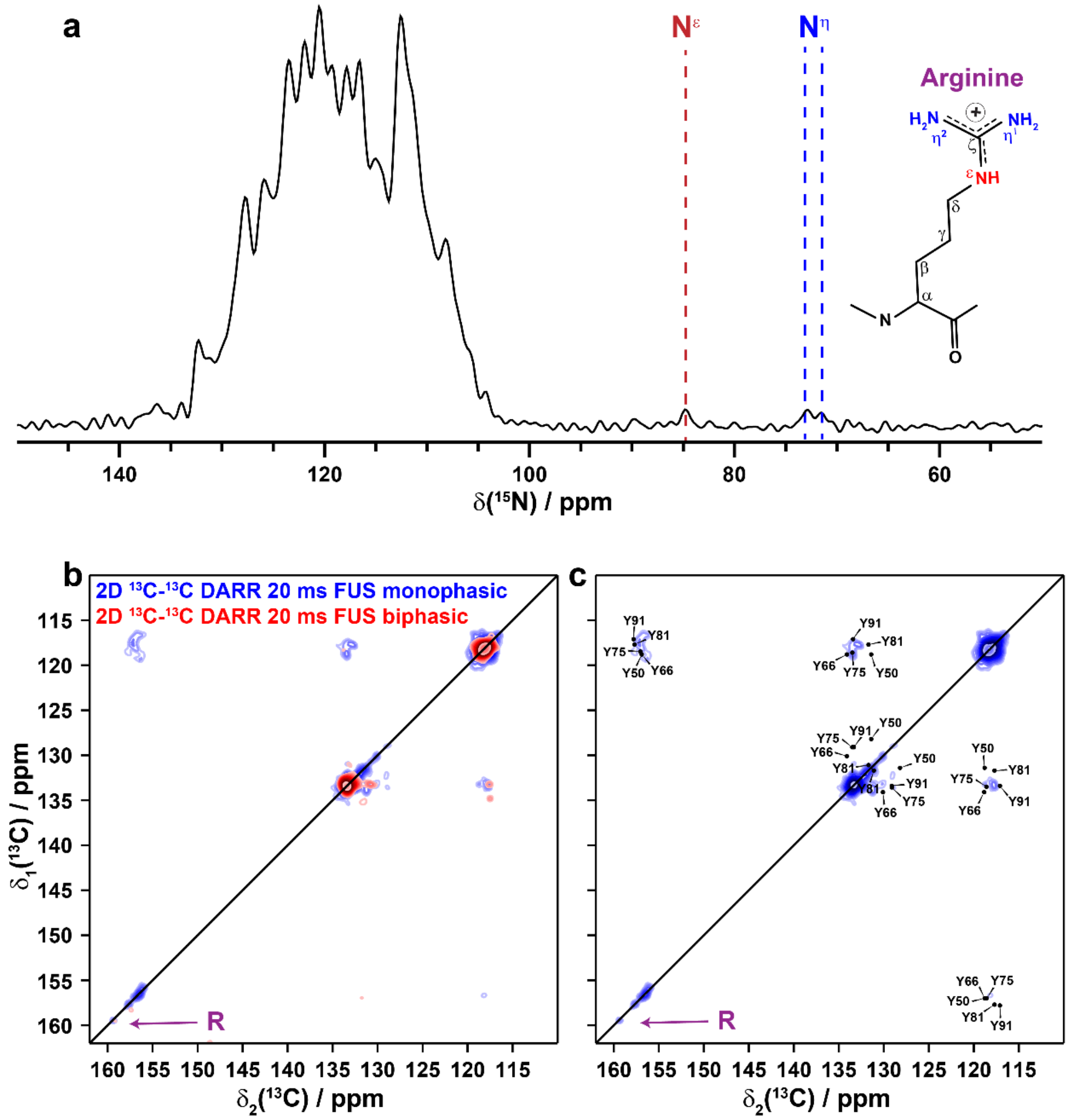

Supplementary Fig. 4: a) 1D ^{15}N CP MAS spectrum of monophasic FUS NTD. The signals arising from the N^{ε} and N^{η} atoms of the arginine side chains are highlighted in red and blue respectively. b) Comparison of the aromatic regions of the 2D DARR spectra recorded on maturated mono- and biphasic FUS-NTD samples. In the monophasic sample, a Cζ arginine side chain diagonal peak highlighted by the purple arrow is observed. c) Aromatic region of the 2D DARR spectrum of the monophasic sample with back-predicted shifts for the FUS fibril core from [4].

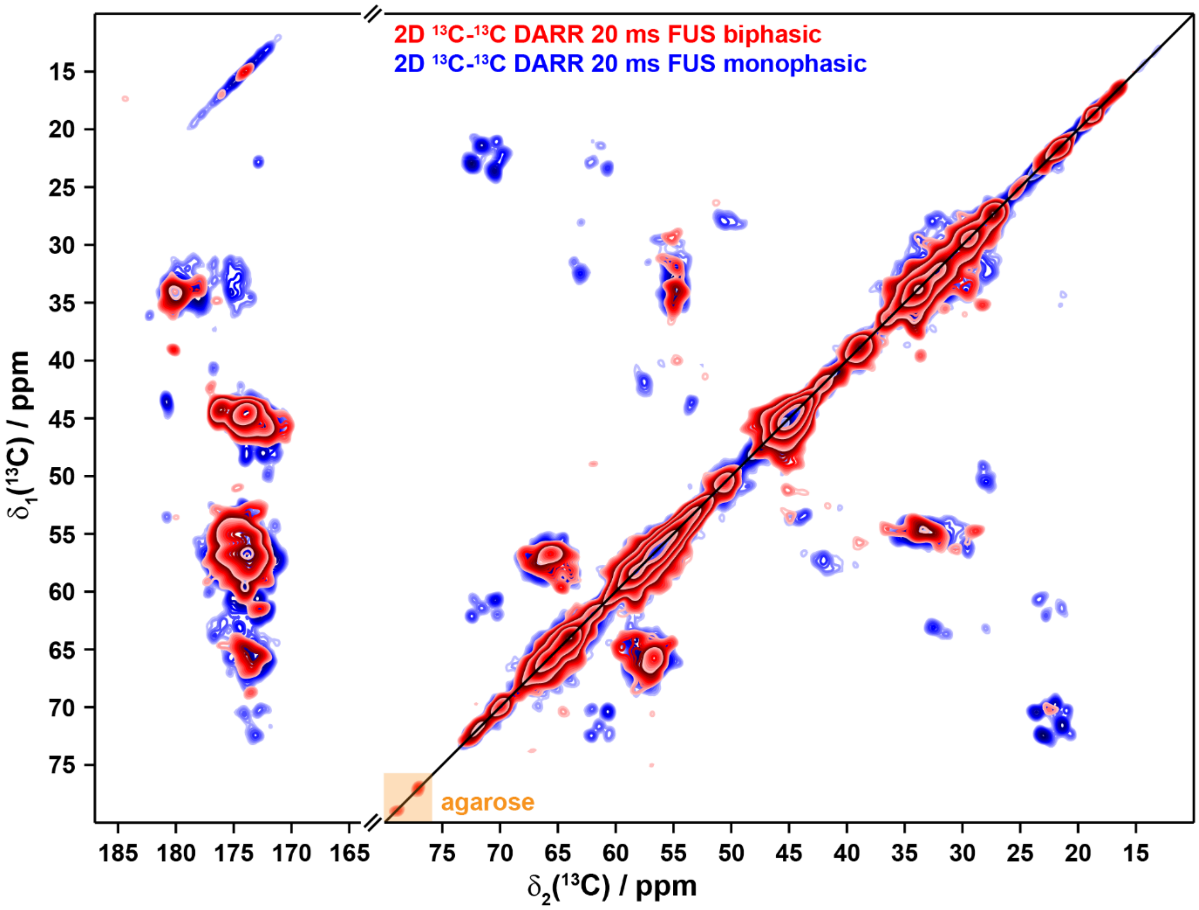

Supplementary Fig. 5: Comparison of 2D DARR spectra of the maturated mono- and biphasic FUS-NTD samples (68 days and 37 days of maturation). The orange box indicates ^{13}C signals originating from the agarose hydrogel matrix of the biphasic sample.
